# Prediction of the optimal depth for superior vena cava cannulae with cardiac computed tomography during minimally invasive cardiac surgery: a prospective observational cohort study

**DOI:** 10.1186/s12871-017-0347-x

**Published:** 2017-04-07

**Authors:** Ji-Hyun Chin, Eun-Ho Lee, Jong-Il Kim, In-Cheol Choi

**Affiliations:** Department of Anesthesiology and Pain Medicine, Asan Medical Center, University of Ulsan College of Medicine, 88, Olympic-ro 43-gil, Songpa-gu, Seoul, 05505 South Korea

**Keywords:** Cardiac computed tomography, Superior vena cava cannulae, Minimally invasive cardiac surgery

## Abstract

**Background:**

The determination of the adequate depth of superior vena cava cannulae during minimally invasive cardiac surgery is important for warranting venous drainage and preventing complications during cardiopulmonary bypass. We investigated whether preoperative cardiac computed tomography might be useful for predicting the optimal depth of superior vena cava cannulae.

**Methods:**

The patients who required superior vena cava cannulation and had cardiac tomographic image among those scheduled to undergo a minimally invasive cardiac surgery were evaluated. The distance between the upper border of the clavicular sternal head and the superior vena cava-right atrium junction was measured on cardiac computed tomography. Equivalence test for the difference between the distance measured on cardiac computed tomography and the distance verified by surgeon’s direct inspection in the surgical field was performed. The range −1 cm to 1 cm was predefined as an equivalence region. In addition, the distances between the upper border of the clavicular sternal head and the carina level on chest radiography were measured to compare the relative position of carina with regard to the superior vena cava-right atrium junction.

**Results:**

A total of 46 patients were evaluated. The distance from the upper border of the clavicular sternal head to the superior vena cava-right atrium junction measured on cardiac computed tomography and the distance verified by surgeon’s inspection was equivalent, with the 95% confidence interval for the mean difference within the equivalence region (0.05–0.52, *P* < 0.0001). The carina level on chest radiography was found at least 2 cm above the superior vena cava-right atrium junction in all patients.

**Conclusions:**

Preoperative cardiac computed tomography might be valuable for predicting the adequate depth of superior vena cava cannulae. Additionally, the carina on chest radiography might indicate a useful landmark for proper position of central venous catheter.

**Trial registration:**

This study has been registered at Clinical Research Information Service on 6 July 2012 (KCT0000477).

**Electronic supplementary material:**

The online version of this article (doi:10.1186/s12871-017-0347-x) contains supplementary material, which is available to authorized users.

## Background

Minimally invasive robot-assisted cardiac surgery has several advantages over conventional surgery, such as a decreased risk of infection and postoperative pain, that have led to an expansion of its use in cardiac surgery. To reduce the surgical incision size in minimally invasive cardiac surgery, percutaneous insertion of superior vena cava (SVC) cannulae via the internal jugular vein is necessary for venous drainage during cardiopulmonary bypass. SVC cannulae must be placed at an adequate depth, because a SVC cannula that is too deep increases the risk of cardiac perforation, impedes surgical field, and causes venous air lock in the bypass circuit [1–4], whereas a SVC cannula that is too shallow increases the risk of microemboli and blood trauma [5, 6]. Transesophageal echocardiography has been reported to be useful at the time of cannula insertion to ensure the adequate positioning of SVC cannulae [4].

Several imaging tools, including echocardiography and cardiac computed tomography (CT), have been used to assess the functional and anatomical abnormalities of a cardiac disease prior to cardiac surgery. Cardiac CT has been suggested to have the potential to visualize coaptation failure of mitral leaflets [7], and may become an imaging tool that provides an exact assessment of the function of the valves [8]. Hence, preoperative cardiac CT could be used in patients who undergo cardiac surgery. In addition to its original role in diagnosis, numerous cardiac CT-obtained measurements, such as length or width, of structures adjacent to the heart might enable clinicians to predict the adequate size or depth of an insertion device.

We aimed in our current study to investigate whether preoperative cardiac CT could be used to accurately predict SVC cannulae depth by comparing measurements taken in this way with those obtained by direct inspection of the surgical filed by an experienced cardiac surgeon in patients undergoing minimally invasive robot (DaVinci®)-assisted cardiac surgery. In addition, we evaluated whether other variables, such as patient’s demographic data or results obtained by commonly performed chest radiography, might provide valuable information for predicting or confirming an optimal depth of central venous catheters before and after insertion.

## Methods

### Patients

The study protocol was approved by the Institutional Review Board of the Asan Medical Center (AMC IRB 2012–0347), and written informed consent was obtained for each patient. The study was registered at the Clinical Research Information Service (KCT0000477). Patients who were scheduled to have minimally invasive robot-assisted cardiac surgery requiring SVC cannulation for venous drainage during cardiopulmonary bypass and who had preoperative cardiac CT image were enrolled. Patients younger than 20 years and undergoing emergency surgery were excluded.

### SVC cannulation

SVC cannulation followed induction of general anesthesia. To insert SVC cannulae into the right internal jugular vein, patients were placed in a supine 20° Trendelenburg position with the head rotated 45° to the left. After antiseptic skin preparation and sterile draping, SVC cannulation was performed based on our institution’s protocol. A 17 or 21 Fr cannula was inserted when the body surface area of the patient was < 1.875 m^2^, or ≥ 1.875 m^2^, respectively. SVC cannulae were prepared in heparin (25,000 IU) diluted in normal saline (1000 ml). An ultrasound (M-Turbo®, Fujifilm SonoSite Inc., USA) with a linear probe (6–13 MHz) was used. The ultrasound probe, covered with a sterile sleeve containing sterile ultrasound gel, was placed at the apex of the triangle formed between the two heads of the sternocleidomastoid muscle and the clavicle. The internal jugular vein and common carotid artery were visualized, with the vein identified by relative anatomic position and compressibility. After a flashback of dark venous blood was noted in the syringe, the standard Seldinger technique was followed for SVC cannulae insertion with intravenous administration of 30 IU/kg heparin. SVC cannulae were inserted at the predetermined depth as mentioned below. Chest radiography with an antero-posterior projection (CXR-AP) was performed in the supine position with the patient’s head and neck in the neutral position after SVC cannula insertion. For surgery, intercostal ports were created to allow access of the camera and robotic instruments into the thoracic cavity. After opening the pericardial sac, the position of the SVC cannula tip was examined by an experienced cardiac surgeon. A cardiac surgeon palpated the SVC cannula tip in place and then determined the distance from the SVC cannula tip to the SVC-right atrium (RA) junction by using a sterile disposable ruler. When the cardiac surgeon could not verify the position of the SVC cannula tip because the SVC cannula tip was placed outside the pericardium, the SVC cannula tip was considered to be positioned 3.5 cm above the SVC-RA junction.

### Determination of the optimal depths of SVC cannulae using cardiac CT

**Fig. 1:**
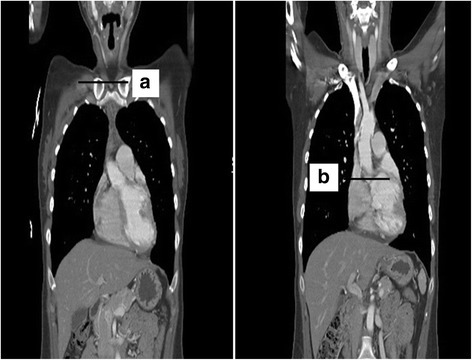
Coronal planes of cardiac computed tomography showing the level of the upper border of the sternal clavicle (**a**) and the level of the junction between the superior vena cava and right atrium (**b**)

To determine the adequate depth of SVC cannula insertion, two lengths were measured: the shortest straight distance between the insertion point of the needle and the level of the upper border of the clavicular sternal head using a sterile disposable ruler, and the distance between the upper border of the clavicular sternal head and the SVC-RA junction in the coronal planes of the cardiac CT. The starting point of the line was fixed at the upper border of the clavicular sternal head in one plane; the line was then continued to another plane showing the SVC-RA junction to end at the SVC-RA junction followed by drawing a line to SVC-RA junction (Fig. 1). Finally, the SVC cannula was inserted to a depth that was determined by the sum of the two measurements minus 1.0–1.5 cm, when surgery involving only the left side of the heart was scheduled, or 2.0–3.0 cm, when surgery involving the right side of the heart was scheduled, as appropriate. The corrected distance from the upper border of the clavicular sternal head to SVC-RA junction was defined as the distance that was adjusted from the measured distance on cardiac CT by the surgeon’s measurement in the surgical field. Here, the surgeon’s measurement was the distance from the SVC cannula tip to the SVC-RA junction in the surgical field, as mentioned above. For example, if the distance from the upper border of the clavicular sternal head to the SVC-RA junction on cardiac CT was 10.0 cm with the SVC cannular tip being planned to be placed 2.0 cm above the SVC-RA junction, and the SVC cannular tip was verified as being positioned 3.0 cm above the SVC-RA junction by the cardiac surgeon, then the corrected distance from the upper border of the clavicular sternal head to the SVC-RA junction was 11.0 cm. The distance between the upper border of the clavicular sternal head and the SVC-RA junction on cardiac CT was measured independently by two investigators. The measurement of the first investigator was used in the analysis.

### Post-hoc analysis of the usefulness of chest radiography for determining the depth of a central venous catheter

The distances between the upper border of the clavicular sternal head and the carina level on preoperative chest radiography with a postero-anterior projection (CXR-PA) and postoperative CXR-AP were measured to compare with the corrected distance between the upper border of the clavicular sternal head and the SVC-RA junction.

### Statistical analysis

Continuous data are expressed as the mean ± standard deviation, and categorical data as number and percentage. A power analysis based on our pilot study data suggested that a minimum sample size of 42 patients would be required to detect a 1.0 cm difference in means (standard deviation: 1.4 cm) for the distance from the upper border of the clavicular sternal head to SVC-RA junction measured on cardiac CT and that verified by surgeon’s inspection with a power of 90% at the *P* < 0.05 of significance. Expecting a dropout rate of about 10%, we aimed to enroll 46 patients.

Equivalency of the measured distance between the upper border of the clavicular sternal head and the SVC-RA junction on cardiac CT compared with the corrected distance determined by the surgeon’s direct inspection was assessed by using the two one-sided tests procedure of Schuirmann [9], which claims equivalency if two simultaneous individual null hypotheses are rejected: first, that the difference is less than the lower bound of a predetermined equivalency interval, and second, that the difference is greater than the upper bound of the equivalency interval. The significance level was 0.05. Using the methods of Schuirmann, this procedure led to the alternative hypothesis of equivalence being accepted if each individual one-sided test was significant at the 0.025 criterion. In the present study, the margin of equivalence was 1 cm and the range −1 cm to 1 cm was predefined as an equivalence region. Reliability analysis of the interobserver agreement for the two measurements was done by calculating a two-way mixed model of intraclass correlation coefficient [10]. A kappa of 0.81–1.00 indicates almost perfect agreement, 0.61–0.80 indicates substantial agreement, 0.41–0.60 indicates fair agreement, 0.21–0.40 indicates slight agreement, and < 0.20 indicates poor agreement [10].

In post-hoc analysis, the Bland-Altman plot of comparison analysis was used to compare the distance from the upper border of the clavicular sternal head to the carina on chest radiography with the corrected distance from the upper border of the clavicular sternal head to the SVC-RA junction. In addition, the correlation between the corrected distance and patient demographic data (height, weight and age) and the correlation between the corrected distance and the difference between the corrected distance and the distance from the upper border of the clavicular sternal head to the carina level on CXR were evaluated by using a Pearson’s or Spearman’s correlation test, as appropriate.

A *P* value < 0.05 was considered statistically significant. All statistical analysis was performed using SAS software version 9.1 (SAS Institute, Cary, NC).

## Results

A total of 46 patients were studied, and those characteristics are shown in Table 1. Of a total of 46 patients, 24 underwent cardiac surgery involving the left side of the heart exclusively, whereas 22 underwent cardiac surgery involving the right side of the heart surgery, regardless of the left side. The interobserver agreement in the measurement of the distance between the upper border of the clavicular sternal head and the SVC-RA junction on cardiac CT was almost perfect, as shown by an intraclass correlation coefficient of 0.91 (95% confidence interval [CI]: 0.75–0.95).

**Table 1:** Patient characteristics

Total patients number (n)	46
Age (years)	51.5 ± 11.6
Sex (M/F)	27/19
Weight (kg)	65.3 ± 12.2
Height (cm)	164.9 ± 8.6
Type of surgery	
Surgery involving only the left side of the heart	24
MVP	21
Myxoma removal	3
Surgery involving the right side of the heart	22
MVP+ TAP	8
MVR + TAP	1
MVP + ASD closure	2
ASD closure	6
ASD closure + TAP	2
TVP	3

Values are expressed as mean ± SD or number. *MVP* mitral valvuloplasty, *TAP* tricuspid annuloplasty, *MVR* mitral valve replacement, *ASD* atrial septal defect, *TVP* tricuspid valvuloplasty

### Equivalency of the distance measured on cardiac CT compared with that verified by direct surgeon’s inspection

The distance from the puncture point to the upper border of the clavicular sternal head was 6.0 ± 0.9 cm (range, 4.0–8.0). The distances from the upper border of the clavicular sternal head to the SVC-RA junction measured on cardiac CT and that verified by the surgeon’s inspection were 11.1 ± 1.4 cm (range, 8.3–13.9) and 11.4 ± 1.4 cm (range, 8.3–14.4), respectively (Table 2), and they did not show significant difference (*P* = 0.334). In 39 patients (84.8%), the difference between the distance measured on cardiac CT and that verified by surgeon’s inspection was from −1 cm to 1 cm (Table 3). It is observed that the distance from the upper border of the clavicular sternal head to SVC-RA junction measured on cardiac CT and that verified by surgeon’s inspection was significantly equivalent, as the 95% CI for the mean difference lay entirely within the equivalency interval (95% CI for the mean difference 0.05–0.52, *P* < 0.0001).

**Table 2:** Measurements on cardiac CT, on chest radiography, or by surgeon’s inspection

Measurements	Values (cm)
Distance from the skin puncture point to the upper border of the clavicular sternal head	6.0 ± 0.9
Distance from the upper border of the clavicular sternal head to the SVC-RA junction on cardiac CT	11.1 ± 1.4
Distance from the upper border of the clavicular sternal head to the SVC-RA junction by surgeon’s inspection (the corrected distance)	11.4 ± 1.4
Distance from the upper border of the clavicular sternal head to carina on CXR-PA	7.1 ± 1.0
Distance from the upper border of the clavicular sternal head to carina on CXR-AP	7.0 ± 1.1
Difference between the corrected distance and (a)	4.3 ± 0.9
Difference between the corrected distance and (b)	4.4 ± 0.9

Values are expressed as mean ± SD. *SVC* superior vena cava, *RA* right atrium, *CT* computed tomography, *CXR-PA* chest radiography with postero-anterior projection, *CXR-AP* chest radiography with antero-posterior projection, (a) distance from the upper border of the clavicular sternal head to carina on CXR-PA, (b) distance from the upper border of the clavicular sternal head to carina on CXR-AP

**Table 3:** Differences between the predetermined and verified distance from SVC cannula tip to SVC-RA junction

Difference	Number (%)
−2 < D < −1	1 (2.2)
−1 ≤ D ≤ 1	39 (84.8)
−1 ≤ D < 0	10 (21.7)
D = 0	16 (34.8)
0 < D ≤ 1	13 (28.3)
1 < D ≤ 2	2 (4.3)
2 < D ≤ 3	4 (8.7)

Values are expressed as number (percentage). *SVC* superior vena cava, *RA* right atrium, *D* difference between the predetermined distance from the SVC cannula tip to the SVC-RA junction and the verified distance from the SVC cannula tip to the SVC-RA junction determined by a surgeon

#### Relationship between the distance from the upper border of the clavicular sternal head to the carina on chest radiography and the corrected distance from the upper border of the clavicular sternal head to the SVC-RA junction

The distances from the upper border of the clavicular sternal head to the carina on preoperative CXR-PA and postoperative CXR-AP are shown in Table 2. The mean difference between the distance from the upper border of the clavicular sternal head to the carina on CXR-PA and the corrected distance from the upper border of the clavicular sternal head to the SVC-RA junction was 4.3 cm (95% CI: 4.1–4.6 cm) and 95% limits of agreement were 2.5–6.2 cm. In addition, the mean difference between the distance from the upper border of the clavicular sternal head to the carina on CXR-AP and the corrected distance from the upper border of the clavicular sternal head to the SVC-RA junction was 4.4 cm (95% CI: 4.1–4.7 cm) and the 95% limits of agreement were 2.6–6.2 cm.

As shown in Table 4, the carina levels on CXR-PA and CXR-AP were always located at least 2 cm above the SVC-RA junction. The distance from the carina to the SVC-RA junction was equal to or more than 3 cm and less than 5 cm in 32 patients on both CXR-PA and CXR-AP and equal to or more than 5 cm in 11 patients on CXR-PA and 13 patients on CXR-AP (Table 4).

**Table 4:** Differences between the corrected and measured distance from upper border of clavicular head to carina

Difference	CXR-PA	CXR-AP
0 ≤ D _jx to carina_ < 2	0 (0%)	0 (0%)
2 ≤ D _jx to carina_ < 3	3 (6.5%)	1 (2.2%)
3 ≤ D _jx to carina_ < 4	11 (23.9%)	14 (30.4%)
4 ≤ D _jx to carina_ < 5	21 (45.7%)	18 (39.1%)
5 ≤ D _jx to carina_ < 6	10 (21.7%)	12 (26.1%)
6 ≤ D _jx to carina_	1 (2.2%)	1 (2.2%)

Values are expressed as number (percentage). *SVC* superior vena cava, *RA* right atrium, *CXR-PA* chest radiography with postero-anterior projection, *CXR-AP* chest radiography with antero-posterior projection, *D*_*jx to carina*_ difference between the corrected distance from the upper border of the clavicular sternal head to the SVC-RA junction and the measured distance from the upper border of the clavicular sternal head to the carina on chest radiography

The corrected distance from the upper border of the clavicular sternal head to the SVC-RA junction was significantly correlated with the distance from the upper border of the clavicular sternal head to the carina on chest radiography (CXR-PA: *r* = 0.76, *P* < 0.0001; CXR-AP: *r* = 0.76, *P* < 0.0001). The corrected distance from the upper border of the clavicular sternal head to the SVC-RA junction was not correlated with height (*r* = 0.15, *P* = 0.34), weight (*r* = 0.14, *P* = 0.35) or age (*r* = 0.10, *P* = 0.53), and did not differ according to sex (11.7 ± 1.5 in male vs. 11.0 ± 1.2 cm in female, *P* = 0.063).

## Discussion

We have shown that cardiac CT is a valuable tool for predicting the optimal depth of SVC cannulae by comparing the equivalence of the distance measured on cardiac CT and the distance verified by a surgeon’s direct inspection. In addition, we found that the carina levels on chest radiography were always located at least 2 cm above the SVC-RA junction, indicating the usefulness of CXR-PA in predicting the depth of a central venous catheter and of CXR-AP in confirming it.

It is impossible to overemphasize the importance of an adequate positioning of SVC cannulae for preventing complications, including cardiac perforation, an obscured surgical field, venous air lock in the bypass circuit, and microemboli, all of which can be caused by an inadequately positioned SVC cannula [1–6]. Particularly during minimally invasive cardiac surgery, the inherent characteristics of the percutaneous catheter, such as a longer length and smaller internal diameter compared with standard cardiopulmonary bypass cannulae, reduce venous drainage efficiency during bypass surgery [11]. Hence, adequate positioning of SVC cannulae may become a substantially more important issue as use of minimally invasive cardiac surgery increases. Transesophageal echocardiography has been reported to be the standard method for the optimal placement of SVC cannulae in minimally invasive cardiac surgery [4, 12]. Transesophageal echocardiography-guided cannulation is performed via real-time imaging and thus instantly ascertains the position of SVC cannulae. Previous studies confirmed that transesophageal echocardiography could be used to guide the successful placement of SVC cannulae, but these reports did not provide supplementary information regarding prediction of SVC cannula depth before insertion. Our present study was conducted to determine whether a diagnostic imaging tool, other than transesophageal echocardiography, that is routinely performed before cardiac surgery could have another use, such as in the prediction of an adequate SVC cannula depth in the operating room. We determined that the measurement obtained by on cardiac CT and that verified by the surgeon’s direct inspection were comparable, indicating the usefulness of cardiac CT for predicting the depth of SVC cannula, in addition to its diagnostic role. Therefore, the final depth for SVC cannulae to be inserted might be the sum of the distance from the upper border of the clavicular sternal head to the SVC-RA junction which is measured in cardiac CT and the distance from the puncture point to the upper border of the clavicular sternal head which is dependent on the insertion point. To our knowledge, our present study is the first to investigate the usefulness of this imaging tool for predicting an adequate depth of an externally inserted catheter by comparing with the measurements obtained from direct inspection. The depth of SVC cannulae determined by using cardiac CT might additionally support the adequate positioning of SVC cannulae tips.

Post-hoc analysis showed that the carina level on chest radiography, which could be easily identified and limited parallax effect [13], was a reliable landmark for determining an adequate depth of a central venous catheter. Several studies have already addressed that the carina level was slightly above the pericardial reflection in dissected cadaver [14–16]. Nevertheless, none of these studies have proposed clinically useful radiographic landmark that corresponds the optimal depth of a central venous catheter, because they only found an anatomical relationship relative to the carina level in cadavers, without validation of the carina level in clinically relevant radiographic data. The present study addressed the position of an anatomic landmark, or the carina level, on chest radiography relative to that of an actual anatomic structure, or the SVC-RA junction, in adult patients. The tip of a central venous catheter is recommended to be positioned in the SVC and outside the pericardial sac to prevent serious complications such as cardiac tamponade. As the pericardial reflection is as high as 2 cm above the SVC-RA junction [17], the optimal depth of the central venous catheter is 2–3 cm above the SVC-RA junction. Our study showed that the carina level was always at least 2 cm above the SVC-RA junction in all studied patients, indicating the valuable role of the carina as a landmark on chest radiography. Furthermore, we found that the carina level would be 2.5–6.2 cm above SVC-RA junction in about 95% patients. Therefore, we can confirm the findings of previous cadaver studies and suggest that the carina level on CXR-PA and CXR-AP is a useful landmark for predicting the adequate depth of a catheter before insertion and for verifying the adequate depth after insertion.

We further found that the distance from the upper border of the clavicular sternal head to the SVC-RA junction did not correlate with height, weight, sex, or age. This result supports a previous study in which the lengths measured in the thorax, including the intrapericardial part of the SVC and the distance between the carina and the pericardial reflection, did not correlate with height in fresh cadavers [15], and disproves the clinical practice that a depth of a central venous catheter was adjusted according to patient height.

Our study has following limitations. First, we did not evaluate how far the SVC cannula tip was positioned from the pericardial reflection, because it was impossible to discern the distance between the SVC cannula tip and the pericardial reflection due to the restricted surgical field. Further study is required to identify it in cardiac surgical patients undergoing open sternotomy. Second, our result could not induce the preferential use of cardiac CT to transesophageal echocardiography when determining the optimal depth for SVC cannulae, because our study was not designed to examine the superiority of cardiac CT to transesophageal echocardiography. Our result might show another helpful aspect of using cardiac CT image besides its primary role for diagnosing cardiac disease, when cardiac CT image was available in minimally invasive cardiac surgical patients. Third, venous drainage via SVC cannulae during cardiopulmonary bypass was not objectively evaluated.

## Conclusions

Cardiac CT may provide useful information for predicting the adequate depth of SVC cannulae for venous drainage during cardiopulmonary bypass. This was driven from compelling evidence provided by our comparisons between cardiac CT-obtained measurements and that obtained via direct inspection of anatomical structures in the surgical field. In addition, the carina level may be confirmed to be worthy landmark on chest radiography for the placement of central venous catheters in the SVC, which is based on the finding that the carina level on chest radiography was at least 2 cm above the SVC-RA junction in all patients.

## Additional file

Additional file 1: The dataset. This file shows the dataset of our study. (XLSX 14 kb)

## Abbreviations

CI: Confidence interval
CT: Computed tomography
CXR-AP: Chest radiography with an antero-posterior projection
CXR-PA: Chest radiography with a postero-anterior projection
RA: Right atrium
SVC: Superior vena cava

## References

[1] Chabanier A, Dany F, Brutus P, Vergnoux H (1988). Iatrogenic cardiac tamponade after central venous catheter. Clin Cardiol.

[2] Collier PE, Ryan JJ, Diamond DL (1984). Cardiac tamponade from central venous catheters: report of a case and review of the English literature. Angiology.

[3] Defalque RJ, Campbell C (1979). Cardiac tamponade from central venous catheters. Anesthesiology.

[4] Lee YK, Sim JY, Seo JW, Choi IC, Hahm KD, Choi JW (2010). Optimal placement of a superior vena cava cannula in minimally invasive robot-assisted cardiac surgery. Circ J.

[5] Win KN, Wang S, Undar A (2008). Microemboli generation, detection and characterization during CPB procedures in neonates, infants, and small children. ASAIO J.

[6] Martin R, McKenty S, Thisdale Y, Lavallee P, Teijeira J, Bonneau D (1989). Hemolysis during cardiopulmonary bypass. J Cardiothorac Anesth.

[7] Alkadhi H, Wildermuth S, Bettex DA, Plass A, Baumert B, Leschka S (2006). Mitral regurgitation: quantification with 16-detector row CT--initial experience. Radiology.

[8] Schroeder S, Achenbach S, Bengel F, Burgstahler C, Cademartiri F, de Feyter P (2008). Cardiac computed tomography: indications, applications, limitations, and training requirements: report of a Writing Group deployed by the Working Group Nuclear Cardiology and Cardiac CT of the European Society of Cardiology and the European Council of Nuclear Cardiology. Eur Heart J.

[9] Schuirmann DJ (1987). A comparison of the two one-sided tests procedure and the power approach for assessing the equivalence of average bioavailability. J Pharmacokinet Biopharm.

[10] Landis JR, Koch GG (1977). The measurement of observer agreement for categorical data. Biometrics.

[11] Jegger D, Tevaearai HT, Horisberger J, Mueller XM, Boone Y, Pierrel N (1999). Augmented venous return for minimally invasive open heart surgery with selective caval cannulation. Eur J Cardiothorac Surg.

[12] Wang Y, Gao CQ, Wang G, Wang JL (2012). Transesophageal echocardiography guided cannulation for peripheral cardiopulmonary bypass during robotic cardiac surgery. Chin Med J (Engl).

[13] Yoon SZ, Shin JH, Hahn S, Oh AY, Kim HS, Kim SD (2005). Usefulness of the carina as a radiographic landmark for central venous catheter placement in paediatric patients. Br J Anaesth.

[14] Schuster M, Nave H, Piepenbrock S, Pabst R, Panning B (2000). The carina as a landmark in central venous catheter placement. Br J Anaesth.

[15] Albrecht K, Nave H, Breitmeier D, Panning B, Troger HD (2004). Applied anatomy of the superior vena cava-the carina as a landmark to guide central venous catheter placement. Br J Anaesth.

[16] Albrecht K, Breitmeier D, Panning B, Troger HD, Nave H (2006). The carina as a landmark for central venous catheter placement in small children. Eur J Pediatr.

[17] Aslamy Z, Dewald CL, Heffner JE (1998). MRI of central venous anatomy: implications for central venous catheter insertion. Chest.

